# Research and Prospect Analysis of Sports Consumption Willingness Based on Public Health Emergencies

**DOI:** 10.3389/fpsyg.2021.792686

**Published:** 2022-01-31

**Authors:** Ziyuan Liu, Rui Guo, Jia Liu, Fan Dong, Yang Shi, Qi Cai

**Affiliations:** ^1^School of Economics and Management, China University of Geosciences, Wuhan, China; ^2^School of Physical Education, China University of Geosciences, Wuhan, China; ^3^School of Science, Minzu University of China, Beijing, China; ^4^School of Foreign Languages, China University of Geosciences, Wuhan, China

**Keywords:** novel coronavirus, characteristics of sports consumption, structural equation model, consumption intention, SOR

## Abstract

In 2020, the sudden outbreak of coronavirus disease 2019 (COVID-19) has had a great impact on the health and life of people all over the world, and the sports industry is facing unprecedented challenges due to its participation and strong clustering. Based on the questionnaire survey, literature analysis, and other research methods, this study introduces the stimulus-organism-response (SOR) theory, takes the sports and consumption of Kunshan citizens as the research subject, and draws lessons from the structural equation model (SEM) to build a theoretical model of sports consumption characteristics and future consumption willingness. The results of empirical analysis show that physical sports consumption has been greatly affected by the epidemic, but because people realize the importance of sports, the willingness of residents to consume sports increases, and the venue and other factors affect the ornamental and participating sports consumption willingness decreases. At the same time, the restrictive factors, such as lower educational background, increased age, and lack of time, make the sports consumption willingness of this characteristic group significantly lower than that of other citizens. This study puts forward some suggestions for relevant government departments to improve the sports consumption willingness of citizens. In order to expand the development prospect of sports industry from a long-term perspective, it can provide reference for the development of sports consumption.

## Introduction

In December 2019, patients with unexplained pneumonia appeared in some medical institutions in Wuhan. On 12 January 2020, was named by the World Health Organization (WHO) as a 2019 novel coronavirus. Faced with this unexpected situation, various industries in the world are facing different degrees of impact and blow, including the sports industry. Affected by the pneumonia epidemic in coronavirus disease 2019 (COVID-19), many important sports events were canceled or delayed (Mingzhi et al., 2020). How to face this challenge clearly and rationally, how to get through the temporary difficult period smoothly, and how to turn disadvantages into advantages soon after the outbreak is over will become an urgent problem for enterprises in relevant departments.

The wide spread of the fitness video of Academician Zhong Nanshan has aroused the desire and demand of people for physical fitness. Since February 2020, the State Sports General Administration has held a videoconference on the prevention and control of the epidemic situation in the national sports system and the key sports work in 2020 (State Physical Cultural Administration, 2020a,b), and sports enterprises all over the country have issued policies to resume work and resume production. In the long run, the sports consumption market has broad prospects for development, and sports consumption will usher in explosive growth. At present, there are minimal researches on the sports consumption willingness of residents during the epidemic period, and there is still room for further research in this field. On the basis of sorting out the literature on sports consumption characteristics, participation, and willingness to spend, this study explores the influence mechanism of the sports consumption characteristics of residents on the sports consumption willingness under the epidemic based on the psychology stimulus-organism-response (SOR) theory, uses the software SPSS 20.0 and AMOS 22.0 to build the structural equation modeling (SEM), and makes an empirical analysis on the data from the questionnaire survey of Kunshan residents, hoping to provide practical value for sports industry-related enterprises.

## Review of Related Literature

### Sports Response to the Epidemic-Related Research Brief

The sudden pneumonia epidemic has made the spread and prevention of the virus become the focus of academic and social attention. Various studies have also shown that enhancing immunity is the foundation of fighting viruses. People began to realize the importance of sports, and it has gradually become a public consensus to enhance physical exercise and improve their immunity. Jianwen and Aihong (2021) pointed out that the health value of physical exercise is coming back. The novel coronavirus epidemic is similar to severe acute respiratory syndrome (SARS). Yalun et al. (2020) believed that according to the judgment of the SARS situation, the awareness of people on physical exercise and consumption behavior have been significantly improved after the epidemic. Qiyuan (2020) found that various sports events and sports performances were canceled or postponed, and gyms and other stadiums were closed, with a negative impact on household sports consumption and sports market in the short term, while bringing new development opportunities to the sports industry by affecting consumer concepts in the long run. Jun and Xiangyang (2021) proposed that the sports industry is an important part of the modern service industry, and finding solutions and strategies to the sports consumption dilemma is crucial to the recovery of the sports industry.

Through the search, it is found that study in the field of sports under the novel coronavirus epidemic mainly concentrates on “physical exercise,” “school physical education,” “sports consumption,” “health awareness,” and “sports psychology” but less supported by the actual data. This study makes an empirical analysis of the data from the survey of Kunshan residents in order to have practical value.

### Overview of Stimulus-Organism-Response Theory

The SOR theory is proposed by the environmental scientists Mehrabian and Russell to explore the influence of external environmental stimuli on individual cognition, emotion, behavior, and physical response. Man and Xiangzhi (2020) noted that S (Stimulus) refers to external factors affecting individuals, O (Organism) refers to the internal state of a person, including sensory, emotional, and cognitive behavior, and R (Response) is the behavioral decision of the organism by integrating external environmental stimuli and internal psychological attitude. Stimuli and responses are connected through a series of intrinsic variables of the organism, which is widely applied to a systematic analysis of human behavioral intentions by focusing on the intrinsic emotional and cognitive factors of people.

Combining the research of previous scholars, Lei and Xiaoqiang (2021) pointed out that the SOR models have been widely used in the study of consumer behavior; Russell and Mehrabian (1999) and Mattila and Wirtz (2001) indicate that consumers develop cognitive, emotional, and physical responses to external stimuli, and these reactions comprehensively affect the actual behavior of consumers; At the same time, Mei et al. (2021) pointed out that the SOR model is an important theoretical perspective to study consumer behavior. Therefore, we believed that the SOR framework model provides a very suitable research perspective and a solid theoretical foundation for the study of sports consumption willingness under the epidemic situation. This study uses the pneumonia epidemic as an external environment S, the public internal cognitive and emotional behavior changes as O, and the future sports consumption willingness as R. It makes up for the lack of interdisciplinary integration research between sports, consumer behavior, and psychology.

### Overview of Structural Equation Models

The SEM can not only deal with the causal relationship between measurable variables but also construct multiple “latent variables” by factor analysis, and it can discuss the complex relationship between latent variables or between latent variables and measurable variables. SEM provides the possibility to measure highly abstract and difficult variables. Due to the abovementioned advantages, SEM shows the uniqueness that other analysis methods do not have, and it is widely used by researchers.

Chunming et al. (2021) discussed the impact of the changes in the behavior demand of the Chinese consumers on the perception of various dimensions of the indicators of the sports industry marketing strategy under the normalization of the epidemic; Weiti and Ke (2021) used the SEM to identify and evaluate risk factors in large sports events; Dongabe (2020) used SEM to discuss the influence of exercise time and behavior control on the improvement of the physical fitness of students. Huiqin (2018) used the SEM to discuss the influence of external exercise support, subjective norms, perceptual value, behavior control, and other factors on the exercise willingness of middle school students in Yangzhou; Daoli (2018) used SEM to explore the influence of sports involvement on conspicuous sports consumption; Haiyan (2015) used SEM to explore the impact of sports events on urban tourism. This study uses SEM to study the impact of sports consumption characteristics on the sports consumption willingness of residents under epidemic.

### Independent Variable “Sports Consumption Characteristics” and Dependent Variable “Sports Consumption Willingness” and Related Research

Sports consumption refers to all personal consumption behaviors related to sports. Wei and Hongchao (2005) believed that the types of sports consumption are divided into three categories, namely, physical, ornamental, and participatory, according to the different ways and functions of sports consumption. Mullet and Karson (1985) noted in the study that consumption willingness is the psychological motivation and position, namely, that willingness to consume sports reflects the possibility that consumers are willing to consume sports-related products or services.

Mingzhi et al. (2020) analyzed the consumption status of different sports consumption characteristics in China under the epidemic. The physical sports consumption of various physical consumption materials was significantly reduced in response to the epidemic prevention and control situation, but at the same time, the online sports purchase increased, especially for home sports equipment. The ornamental sports consumption of the public buying tickets to watch sports events, sports exhibitions, and sports performances is due to the increased risk of personnel gathering and the delay or cancelation of many sports activities. Participatory sports consumption represented by sports fitness, sports tourism, and sports training has been negatively and significantly affected by the epidemic. Different consumption characteristics under the epidemic situation have a significant impact on consumption willingness. Therefore, the following assumptions are put forward:

Hypothesis 1: The characteristics of physical sports consumption positively affect sports consumption willingness under the epidemic.
Hypothesis 2: The characteristics of participatory sports consumption negatively affect sports consumption willingness under the epidemic.
Hypothesis 3: The characteristics of ornamental consumption negatively affect sports consumption willingness under the epidemic.

### Intermediate Variable “Sports Involvement” and Related Research

The significance of introducing intermediary variable research is to help explain the relationship mechanism between the independent variable and the dependent variable. Participation represents an intrinsic mental state variable, and Flynn and Goldsmith (1993) proposed that engagement reflects the high perception and consumption motivation of products or services in a specific environment; Xiaosong and Jihai (2009) believed that engagement has become an important factor on consumer psychological activity and purchase decisions, and Dan (2015) noted that the impact of consumer involvement on repeated purchases has been demonstrated by many scholars. Therefore, this study introduces the intermediary variable of involvement degree.

The essential difference between sports consumption and other types of consumption lies in “sports.” Therefore, when choosing variables, sports elements are essential. Related concepts include sports level, sports consciousness, sports participation, sports habits, and so on. These concepts have a direct impact on the strength of sports consumption consciousness. Wei (2011) introduced consumer involvement into the research of ornamental sports consumers; Daoli (2018) used sports involvement as an intermediary variable to verify that sports involvement has a positive impact on conspicuous sports consumption. Combined with the questionnaire, this study sets the measurement indexes of sports involvement, including sports intensity, time, and frequency. Schiffman and Kanuk (2000) thought that the purchase intention of consumers is the subjective tendency of consumers to consume a certain product, which is closely related to their behavior. Therefore, the following assumptions are put forward:

Hypothesis 4: Physical sports consumption positively affects participation under the epidemic.
Hypothesis 5: Participatory sports consumption negatively affects participation under the epidemic.
Hypothesis 6: Ornamental sports consumption negatively affects participation during the epidemic.
Hypothesis 7: Sports involvement plays an intermediary role between consumption characteristics and consumption expenditure willingness, and involvement positively affects future sports consumption expenditure willingness.

## Research on the Overall Situation of Sports Consumption

### Sample Situation

In consideration of economy and rationality, this study adopts the methods of field investigation, questionnaire (electronic questionnaire and paper questionnaire), interview, and mathematical statistics.

The questionnaire has been collected for more than 1 year, and the respondents are 6,853 Kunshan citizens from 11 government agencies, 53 companies and enterprises, 6 schools, and 37 communities in 3 districts and 8 towns of Kunshan City. A total of 6,220 questionnaires were collected, 585 invalid questionnaires with incomplete answers and non-Kunshan residents were excluded, and the remaining valid questionnaires were 5,635, with an effective rate of 90.5%.

### Variable Selection

We divided the characteristics of independent variable sports consumption into three dimensions: physical consumption, ornamental consumption and participatory consumption, took the involvement as the intermediary variable and the future sports consumption willingness expenditure as the dependent variables, and selected the corresponding observation variables, as shown in Table 1.

**TABLE 1:** Measurement dimensions and observation variables.

Dimension	Number	Observed variables
Physical consumption	SE1	Sportswear, shoes, and hats
	SE2	Fitness equipment, apparatus, and accessories
	SE3	Outdoor sports and leisure products (such as tents, sleeping bags, etc.)
	SE4	Books, newspapers, magazines, audio-visual products
	SE5	Mobile intelligent wearable devices (such as sports bracelet, sports watch, etc.)
Ornamental consumption	FE1	Tickets, transportation, accommodation and other expenses for watching sports games on site
	FE2	Tickets for sports performances and transportation, accommodation and other expenses
	FE3	Tickets, transportation, accommodation and other expenses for on-site Sports Exhibition
	FE4	Cost of watching sports events on site and obtaining sports information (such as membership)
Participatory consumption	AE1	Daily fitness online training, offline membership card fees
	AE2	Outdoor sports and sports tourism consumption
	AE3	Medical consumption of physical rehabilitation (mainly refers to the cost of sports injury protection)
	AE4	Online sports consumption (such as sports app, online marathon, E-sports)
Involvement	CS1	Frequency of physical exercise
	CS2	Time for physical exercise each time
	CS3	The intensity of each physical exercise
Willingness of sports consumption in the future	RP1	Your total expenditure of sports consumption in the future
	RP2	Expenses for buying sports lottery
	RP3	Expenditure on sports training (such as tennis training) for minor children

### Analysis of the Basic Situation of Sports Consumption of Kunshan Citizens

Based on the information and data obtained from the questionnaire, 5,635 valid data were collected, and the basic information of the samples is shown in Table 2.

**TABLE 2:** Basic information of survey samples.

Variable		Number (*N* = 5,635)	Frequency
Gender	Male	2,742	48.66%
	Female	2,893	51.34%
Age	Under 20	48	0.85%
	20–39	3,338	59.24%
	40–59	1,895	33.63%
	60 and above	354	6.28%
Education	Junior high school and below	1,187	21.06%
	High school	1,343	23.83%
	Bachelor	2,973	52.76%
	Master degree or above	132	2.34%
Occupation	Party and government officials	310	5.50%
	Enterprise staff	1,296	23.00%
	Enterprise management and technical personnel	369	6.55%
	Professional and technical personnel of public institutions	1,513	26.85%
	Individual businesses	162	2.87%
	Retired personnel	430	7.63%
	Student	79	1.41%
	Other	1,476	26.19%

**FIGURE 1:**
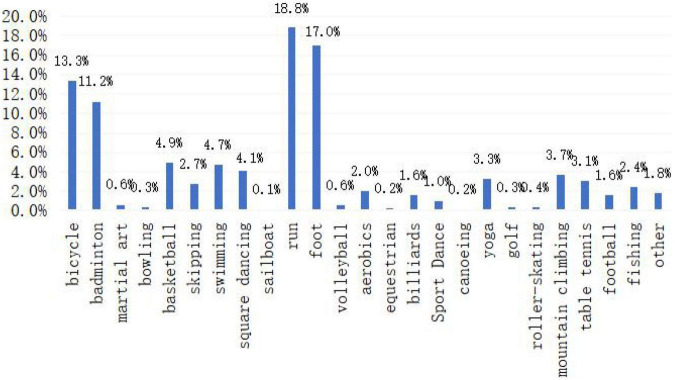
Sports that the respondents often participate in.

The survey results (Figure 1) show that during the epidemic period, Kunshan residents chose running, walking, cycling, and badminton the most. These data are consistent with the statistical results of the “National Fitness Program (2011–2015)” issued by the State Sports General Administration, which shows that physical exercise for the purpose of fighting against the “COVID-19 epidemic” has less to do with the selection of sports events.

**FIGURE 2:**
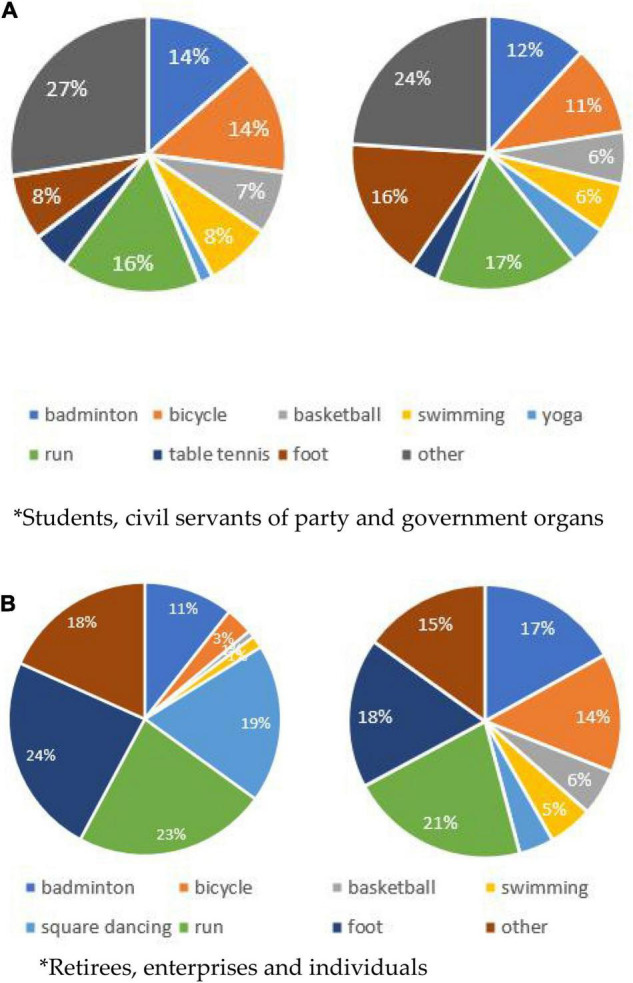
Pie chart of sports events attended by residents of different occupations. **(A)** Students and civil servants of party and government organs. **(B)** Retirees, enterprises, and individuals.

As shown in Figure 2 among them, students prefer running (16%), badminton (14%), and cycling (14%). Public servants of party and government organs prefer running (17%) and hiking (16%). Retired people basically choose to walk (24%), run (23%), and square dance (19%). Enterprises and individuals choose running (21%) and hiking (18%).

**FIGURE 3:**
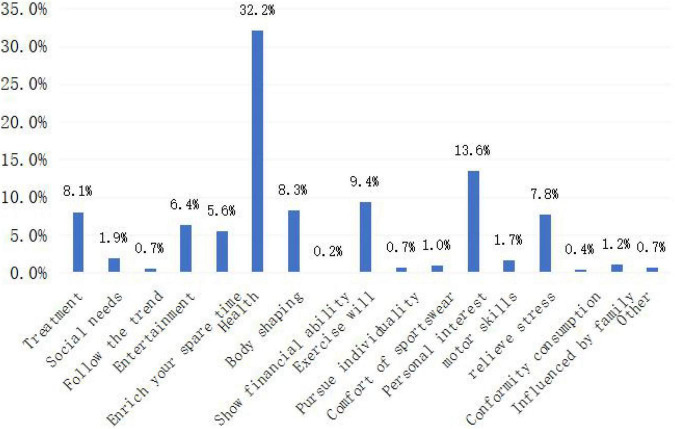
Sports consumption motivation of respondents.

As shown in Figure 3, during the epidemic period, first, most residents spent sports to keep fit. Second, it is due to personal interests, healthcare, and exercise of will. It can be noted from this that the epidemic situation has made people deeply realize the importance of physical exercise to enhance their physical fitness, and physical exercise has become a voluntary lifestyle. This is consistent with Wei and Chaoyang (2003).

### Difference Analysis Under Different Conditions

#### Independent Sample *t*-Test Under the Variable of “Gender”

As shown in Table 3, there are significant differences in scores of variables among residents of different genders. Men are significantly higher than women in terms of time and energy input in operation and willingness to spend sports consumption. This is consistent with the conclusion described by Shanping and Yunbing (2008).

**TABLE 3:** Independent sample *t*-test of gender differences.

Variable	Male (*N* = 2,742)	Female (*N* = 2,893)	*T*
CS	2.450	2.281	10.877**
SE	1.721	1.603	5.860**
AE	1.706	1.600	5.116**
FE	1.439	1.268	9.747**
RP	2.356	2.178	8.972**

**Represents p-value < 0.05.

#### Single-Factor Variance Analysis of “Marital Status”

As shown in Table 4, married residents with two children pay less attention to and invest in sports than those without children.

**TABLE 4:** ANOVA test of “marital status” in each factor.

Marital status	CS		SE		AE		FE		RP	
	Mean	SD	Mean	SD	Mean	SD	Mean	SD	Mean	SD
Unmarried	2.449	0.591	1.688	0.727	1.716	0.764	1.371	0.650	2.036	0.628
Without children	2.406	0.562	1.723	0.764	1.803	0.818	1.449	0.775	2.026	0.657
One child	2.355	0.583	1.639	0.745	1.617	0.758	1.325	0.629	2.296	0.755
Two children	2.310	0.608	1.692	0.787	1.660	0.822	1.376	0.696	2.420	0.771
Other	2.386	0.624	1.540	0.838	1.616	0.887	1.399	0.817	2.168	0.895
F	7.122**		2.693**		6.417**		3.904**		42.619**	

**Represents p-value < 0.05.

#### One-Way Analysis of Variance of “Age”

On the whole, with the increase of age, sports consumption will gradually decline. This shows that compared with the elderly, young people have a stronger sense of sports consumption, participate in more diversified sports, and are willing to spend money on them. However, the elderly are more willing to use simple and easy ways to satisfy their motivation to keep fit. All are shown in Table 5.

**TABLE 5:** ANOVA test of age in each factor.

Age	CS		SE		AE		FE		RP	
	Mean	SD	Mean	SD	Mean	SD	Mean	SD	Mean	SD
Under 20	2.651	0.614	1.844	0.989	1.714	0.878	1.615	0.933	2.163	0.755
20–39	2.307	0.570	1.768	0.754	1.765	0.798	1.391	0.686	2.284	0.745
40–59	2.420	0.590	1.557	0.750	1.540	0.751	1.324	0.641	2.280	0.765
60 and above	2.543	0.689	1.173	0.384	1.170	0.379	1.085	0.296	2.011	0.663
F	31.071**		88.748**		85.183**		27.239**		14.878**	

**Represents p-value < 0.05.

#### Single-Factor Variance Analysis of “Educational Background” and “Monthly Income”

There is a positive relationship between educational level and economic income (Hong and Yang, 2014). Residents with higher educational level usually have higher and more stable economic income, which is shown in Table 6. At the same time, they have a more comprehensive and profound understanding of sports consumption, so their willingness to consume sports is stronger.

**TABLE 6A:** ANOVA test of “education background” on each factor.

A										

Education	CS		SE		AE		FE		RP	
	Mean	SD	Mean	SD	Mean	SD	Mean	SD	Mean	SD
Junior high school and below	2.004	0.468	1.274	0.518	1.279	0.538	1.173	0.442	2.006	0.661
High school	2.387	0.579	1.644	0.705	1.619	0.735	1.407	0.678	2.355	0.74
Bachelor	2.333	0.561	1.798	0.779	1.794	0.817	1.381	0.691	2.316	0.757
Master degree or above	2.431	0.552	2.189	1.063	2.123	0.962	1.699	0.989	2.505	0.886
F	5.761**		173.363**		151.232**		47.300**		64.908**	

**Represents p-value < 0.05.

**TABLE 6B:** ANOVA test of “monthly income” on various factors.

B										

Monthly household income	CS		SE		AE		FE		RP	
	Mean	SD	Mean	SD	Mean	SD	Mean	SD	Mean	SD
Under 6,000	2.406	0.642	1.411	0.61	1.4	0.62	1.236	0.493	2.037	0.681
6,001–10,000	2.305	0.581	1.597	0.689	1.579	0.734	1.326	0.628	2.236	0.713
10,001–16,000	2.345	0.561	1.779	0.744	1.768	0.769	1.37	0.645	2.368	0.75
16,001–20,000	2.429	0.526	1.813	0.785	1.831	0.79	1.41	0.684	2.482	0.762
20,001–26,000	2.454	0.553	2.086	0.966	2.061	0.979	1.634	1.002	2.514	0.833
26,000 above	2.427	0.576	2.241	1.018	2.314	1.016	1.733	1.03	2.653	0.849
F	8.246**		99.540**		103.124**		37.811**		62.152**	

**Represents p-value < 0.05.

## Research on the Influence Mechanism of Sports Consumption

This “Research on the influence mechanism of sports consumption” section mainly analyzes the collected sample data with the help of data analysis software SPSS 20.0 and AMOS 22.0. The specific analysis process is as follows:

### Reliability Test of Scale

In this study, the stability and reliability of the scale were tested by using the corrected item-total correlation (CITC) coefficient and Cronbach’s α value. Cronbach’s α value of the Consumer Consumption Characteristics Scale (Table 7), Involvement Scale, and Consumer Willingness to Spend Future Sports Consumption Scale (Table 8) are all higher than the minimum standard value of 0.60, which indicates that the scales have good reliability.

**TABLE 7:** Reliability analysis of the consumer consumption characteristics scale.

Dimensions		CITC	Item deleted Cronbach’s α	Cronbach’s α
Physical type	SE1	0.429	0.889	0.889
	SE2	0.583	0.882	
	SE3	0.622	0.879	
	SE4	0.599	0.880	
	SE5	0.606	0.880	
Participatory	AE1	0.527	0.886	
	AE2	0.559	0.884	
	AE3	0.595	0.881	
	AE4	0.611	0.880	
Ornamental type	FE1	0.636	0.879	
	FE2	0.685	0.877	
	FE3	0.680	0.877	
	FE4	0.611	0.881	

**TABLE 8:** Reliability analysis.

Variable	Measurement items	CITC	Item deleted Cronbach’s α	Cronbach’s α
Involvement	CS1	0.443	0.663	0.659
	CS2	0.400	0.664	
	CS3	0.689	0.401	
Consumers’ willingness to spend on Sports in the future	RP1	0.490	0.479	0.614
	RP2	0.447	0.561	
	RP3	0.467	0.503	

In the Consumer Consumption Characteristic Scale, the CITC value of SE1 is less than 0.5, so consider eliminating it. After SE1 is eliminated, Cronbach’s α value cannot be significantly improved if any of the remaining 12 measurement items are eliminated. As a result, the questionnaire formed by retaining the remaining 12 measurement items is more accurate.

### Validity Test of Scale

#### Exploratory Factor Analysis

##### Kaiser-Meyer-Olkin and Bartlett Spherical Test

The value of Kaiser-Meyer-Olkin (KMO) was 0.929, and the Bartlett sphere test was significant (Chi-Square = 29728.823, *p* < 0.001). It can be noted that the assumption of independence of each variable is not valid, and the data are suitable for exploratory factor analysis (EFA).

##### Principal Component Extraction

**FIGURE 4:**
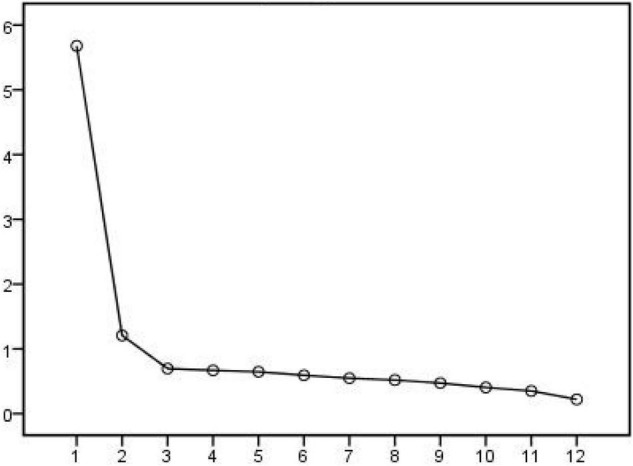
Gravel map.

It can be noted from the broken stone diagram (Figure 4) that the broken line tends to be flat after component 3 and then drops sharply before it, indicating that it is more appropriate to extract the 12 topics of consumer consumption characteristics as three factors.

As shown in Table 9, a total of 3 factors were extracted from 12 topics of consumer consumption characteristics, and the cumulative variance contribution rate was 63.18%, which was more than 60%, and the interpretation degree was ideal.

**TABLE 9:** Total variance explained by each component.

Ingredients	Initial eigenvalue			Extract sum of squares load			Rotation sum of squares loading		
	Total	Variance	Accumulate	Total	Variance	Accumulate	Total	Variance	Accumulate
1	5.679	47.325	47.325	5.679	47.325	47.325	3.126	26.047	26.047
2	1.207	10.057	57.382	1.207	10.057	57.382	2.894	24.116	50.163
3	0.696	5.798	63.180	0.696	5.798	63.180	1.562	13.018	63.180
4	0.668	5.568	68.748						
5	0.646	5.385	74.133						
6	0.592	4.933	79.066						
7	0.547	4.556	83.622						
8	0.519	4.324	87.946						
9	0.472	3.936	91.882						
10	0.404	3.365	95.247						
11	0.350	2.916	98.163						
12	0.220	1.837	100.000						

###### Direct Impact Effect Test

The SEM is adopted for path analysis and inspection, and the established model is shown in Figure 6. The fitting index of this model is as follows: (2/*df* = 23.532; RMSEA = 0.063, less than 0.08). The values of NFI, IFI, goodness of fit index (GFI), and CFI are all greater than 0.90. Overall, the model fits well.

**FIGURE 5:**
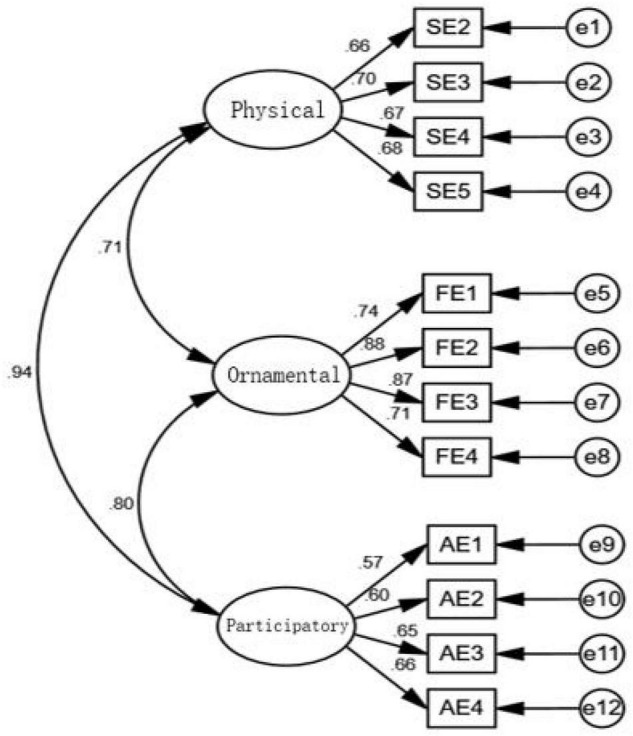
Structural equation model (SEM) of confirmatory factor analysis (CFA) of consumption characteristics (standardized).

**FIGURE 6:**
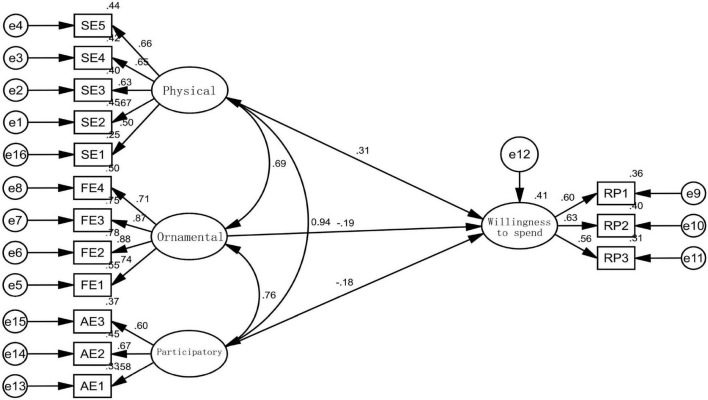
Structural equation model (SEM) (standardization) of the influence of consumption characteristics on future sports expenditure willingness.

####### Mediating Effect Test of Consumer Involvement

Using the Bootstrap Plug-in Process to analyze complex models is a widely used method in recent fields such as psychology and organizational behavior. Rui and Yuhuang (2013) and Rui et al. (2014) introduced its principle and application in detail.

In the path coefficient test of SEM, the p-value represents the significance of mutual influence between paths. If the upper limit and the lower limit of the CI have the same sign *(i.*e., zero is not included), the mediating effect is significant. From Table 14, it can be noted that the degree of involvement has a significant mediating effect in the influence of consumer consumption characteristics on future consumption expenditure intention.

**FIGURE 7:**
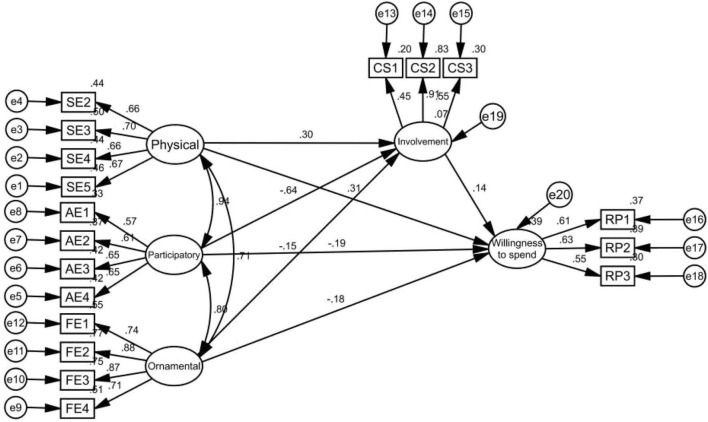
Structural equation model (SEM) (standardized) for the mediating effect test of involvement degree.

With the involvement degree as an intermediate variable (Figure 7, Table 15), the model (2/*df*) decreased by 5.125, RMSEA decreased by 0.007, and NFI, IFI, GFI, CFI, and other indicators reached ideal values, which were very close to 1. Therefore, compared with the original model, the modified model is more effective and has a higher fitting degree.

#### Confirmatory Factor Analysis

Confirmatory factor analysis (CFA) usually selects factor load, mean average variance extraction (AVE), and portfolio reliability (CR) as test marks. In this study, Jietai (2004), and Zhonglin and Jietai (2004) are selected as the criteria for judging each verification index. AVE:


(1) $$\mathrm{AVE}=\frac{\sum \lambda^{2}}{\sum \lambda^{2}+\sum \theta }$$

The formula of combination reliability (CR) is as follows:


(2) $$\mathrm{CR}=\frac{{\left(\sum \lambda \right)}^{2}}{{\left(\sum \lambda \right)}^{2}+\sum \theta }$$

where λ is the normalized factor load and θ is the error variance of the observed variable (θ = 1−λ2).

(1) Consumer consumption characteristics

(1) Structural validity

The model was established in Figure 5. In Table 10, 2/*df* is less than 3; root-mean-square error of approximation (RMSEA) value is 0.068, less than 0.08. The values of normed fit index (NFI), incremental fit index (IFI), TLI, and comparative fit index (CFI) are all greater than 0.90. On the whole, the model of consumer consumption characteristics has good adaptability.

**TABLE 10:** Overall fitting coefficient table.

X2/*df*	RMSEA	NFI	IFI	TLI	CFI
1.402	0.068	0.953	0.955	0.941	0.955

(2) Convergence validity

As shown in Table 11, the factor loads of three dimensions and 12 measurement items of the Consumer Consumption Characteristics Scale are all greater than 0.50, which indicates that each latent variable is highly representative of the subject to which it belongs. Also, the AVE of each latent variable is close to or greater than 0.5, and the CR is greater than 0.70, indicating that convergence validity is good.

**TABLE 11:** Convergence validity test results of consumer consumption characteristics.

Route			Estimate	AVE	CR
SE2	<—	Physical type	0.657	0.457	0.771
SE3	<—	Physical type	0.703		
SE4	<—	Physical type	0.666		
SE5	<—	Physical type	0.678		
FE1	<—	Ornamental type	0.742	0.645	0.878
FE2	<—	Ornamental type	0.876		
FE3	<—	Ornamental type	0.867		
FE4	<—	Ornamental type	0.713		
AE1	<—	Participatory type	0.565	0.484	0.714
AE2	<—	Participatory type	0.605		
AE3	<—	Participatory type	0.647		
AE4	<—	Participatory type	0.658		

(3) Discriminant validity

As shown in Table 12, there is a significant positive correlation between the three latent variables of consumption characteristics (*p* < 0.01), and the absolute values of correlation coefficients are all less than 0.5 and less than the square root of the corresponding AVE. That is to say, there is a certain correlation between the latent variables, and at the same time, there is a certain degree of discrimination between them, which shows that the discrimination validity of the scale data is ideal.

**TABLE 12:** Difference validity test results among variables.

	Physical type	Ornamental type	Participatory type
Physical type	0.457		
ornamental type	0.306***	0.645	
Participatory type	0.453***	0.327***	0.484
Square root of AVE	0.676	0.803	0.696

***Represents p-value < 0.01, and the diagonal line is AVE to evaluate the variance and variation extraction.

In the same way, the SEM of involvement degree and future sports consumption expenditure intention is established.

(2) Involvement degree and future sports consumption expenditure willingness

As shown in Table 13, the factor loads involved in the scale are all close to or greater than 0.50, the AVE is 0.467, and the CR is 0.706.

**TABLE 13:** Convergence validity test results.

Route			Estimate	AVE	CR
CS1	<—	involvement	0.479	0.467	0.706
CS2	<—	involvement	0.932		
CS3	<—	involvement	0.550		
RP1	<—	Willingness to spend	0.514	0.465	0.631
RP2	<—	Willingness to spend	0.629		
RP3	<—	Willingness to spend	0.659		

**TABLE 14:** Standardized bootstrap mediating effect test.

Route	Effect value	SE	Bias-corrected 95%CI			Percentile 95%CI		
			Lower	Upper	*P*	Lower	Upper	*P*
stdIndF1	−0.013	0.016	−0.061	−0.001	0.021	−0.050	−0.001	0.032
stdIndF2	0.028	0.023	0.005	0.088	0.013	0.004	0.084	0.016
stdIndF3	−0.007	0.005	−0.021	−0.001	0.012	−0.019	−0.001	0.017

*stdIndF1, F1-F4-F5; stdIndF2, F2-F4-F5; and stdIndF3, F3-F4-F5.*

**TABLE 15:** Path inspection.

Path	Non-standardized coefficient	Normalization coefficient	S.E.	C.R.	*P*
Physical type → involvement degree	0.196	0.296	0.095	−2.065	0.039
Participatory type → involvement degree	−0.554	−0.637	0.153	3.622	0.000
Ornamental type → involvement	−0.129	−0.146	0.042	−3.072	0.002
Involvement degree → willingness of future consumption expenditure	0.145	0.145	0.023	2.380	0.017

The factor load, AVE, and CR of the future sports consumption expenditure willingness scale are all greater than 0.50.

## Results and Discussion

As a public health emergency, the novel coronavirus epidemic has had a great impact on many industries, as well as the sports industry. Based on sports and consumer behavior, psychology, and marketing, this study constructs SEM to study the influence of sports consumption characteristics and sports consumption involvement degree on the future consumption spending willingness under the background of public health emergencies, drawing the following conclusion:

First, this study tested the direct impact of the three dimensions of sports consumption characteristics (physical, participatory, and ornamental) on the future sports consumption willingness. Through analyzing the SEM, the three-dimensional variables reached the significance level, and the path coefficient and absolute value ranking of consumption characteristics on future consumption willingness were physical (0.31) > ornamental (–0.19) > participatory (–0.18), i.e., Hypotheses 1–3 are verified: indicating that citizens with physical sports consumption characteristics are greatly affected by public health emergencies, but because the awareness of residents of paying attention to life and health is stimulated, and their willingness to make sports consumption is enhanced. However, the ornamental and participating sports consumption is reduced by the venue and other factors, but due to the online and offline characteristics, some residents prefer outdoor sports, such as mountaineering and hiking, with a small population density, so it is less affected by the epidemic.

Second, the study tested the influence of the three dimensions of sports consumption characteristics (physical, participation, and ornamental) on the involvement degree of sports. As a state of psychology, while sudden public health events cause panic to consumers, the public has a more strong awareness of healthcare to enhance the immunity of body through exercise. According to the SEM verification, participatory and ornamental consumption have a negative impact on the involvement of sports, and physical consumption has a positive impact on the involvement of sports. Hypotheses 4–6 that were proposed in this study were verified.

Studies further test the mediation of sports degree. In the path, 95% CI has the same limit (i.e., excluding zero), so sports in sports degree between sports consumption characteristics and future sports consumption will play a significant intermediary role, thus proving that Hypothesis 7 is established.

Residents with different gender, occupation, educational background, marital status, age, and income level have significant differences in sports consumption willingness in all dimensions. The concept of “spending money to buy health” has been accepted by most people, but the formation of the sports consumption concept is also affected by material living standards and cultural development (Hongmei et al., 2010; Shengguo et al., 2016). This fully reflects the contradiction between the pursuit of citizens of quality of life and their limited ability.

## Development Trend and Prospect of Sports Consumption

### Improve the Sports Awareness of Residents and Increase Government Support

The COVID-19 epidemic has greatly improved the awareness of people on sports and health promotion. In this epidemic, the whole society advocates home fitness, and the government and relevant departments can play the role of neighborhood committees. Establishing some small-scale and small-group sports activities can increase sports activities and also avoid large-scale crowd gatherings. At the same time, aiming at citizens of different ages and occupations, different types of activity associations should be established. For example, the elderly prefer the slower-paced sports activities, while the normally busy citizens prefer the relatively lower-intensity sports activities. At the same time, the epidemic situation has also stimulated the scales of small-sized fitness equipment suitable for home use.

### Promote Diversified Integration Among Various Industries and Improve the Industrial Chain

On 18 March 2020, the Ministry of Culture and Tourism held a press conference on “Promoting the resumption of the cultural tourism industry in an orderly manner and actively promoting the potential of consumption replenishment.” Under this mobilization, tourism started a new prelude to resume work and production. All provincial capitals and cities seize the opportunity to improve the industrial chain of the sports tourism industry, promote the continuous integration of sports tourism with clothing, accommodation, transportation, entertainment, and other industries, and realize synergy and development of new consumption. Residents naturally pay monetary costs for sports tourism and promote the development of the sports consumption industry.

### Effective Market Segmentation and Precise Service

Considering low-density participation, scene experience, and non-contact service, we will provide different sports products for different market segments, such as setting up stadiums of different grades. It is to adopt a differentiation strategy to meet the needs of different consumer groups, so as to enhance the sports consumption willingness of residents and obtain the long-term sustainable development benefits.

For example, according to the Tmall 2019 Sports Consumption Trends Report (2019), in terms of sports and fitness, women have also shown unprecedented enthusiasm in recent years, such as yoga based on female groups, and practitioners, in 2014, from 10 million people increased to 20 million people. In 2016, there were 14,000 yoga studios, and by 2018, there were over 30,000 yoga studios. Being keenly aware of market changes and providing accurate services to specific consumer groups will make you stand out and seize market share.

### Sports Enterprises Tend to Be Branded and Digitized

In recent years, the widespread popularity of the Internet has made the public increasingly dependent on the Internet. There is also a new force emerging in the sports industry, such as Keep, Music Carving, Super Orangutan, and other sports fitness apps. After the epidemic, enterprises can make full use of the Internet, break through the time and space constraints, and realize the perfect combination of online and offline sports.

As an offline enterprise, while coping with the crisis and resuming production, it also needs to have an innovative spirit, constantly update its strategic layout, make its service content and products more attractive, and firmly grasp the hearts of consumers. For example, the use of brand membership. Usually, consumers with membership cards have a stronger willingness to consume than other consumers. When the membership system makes the participation of citizens become a stable habit, it will help to increase their sports consumption needs. The participation of one family member in sports will also lead to the participation of other family members. It is still a long-lasting battle to establish brand loyalty in the sports consumption market. Only by finding our own core competitiveness, we can ride the wind and waves in the fierce competition.

## Data Availability Statement

The original contributions presented in the study are included in the article/supplementary material, further inquiries can be directed to the corresponding authors.

## Author Contributions

ZL: conceptualization, methodology, software, formal analysis, resources, data curation, writing–original draft preparation, and project administration. RG and FD: project administration and funding acquisition. JL: writing–review and editing, methodology, and formal analysis. YS: methodology, validation, formal analysis, investigation, and visualization. QC: formal analysis and supervision. All authors contributed to the article and approved the submitted version.

## Conflict of Interest

The authors declare that the research was conducted in the absence of any commercial or financial relationships that could be construed as a potential conflict of interest.

## Publisher’s Note

All claims expressed in this article are solely those of the authors and do not necessarily represent those of their affiliated organizations, or those of the publisher, the editors and the reviewers. Any product that may be evaluated in this article, or claim that may be made by its manufacturer, is not guaranteed or endorsed by the publisher.
